# *Choricystis* and *Lewiniosphaera* gen. nov. (Trebouxiophyceae Chlorophyta), two different green algal endosymbionts in freshwater sponges

**DOI:** 10.1007/s13199-020-00711-x

**Published:** 2020-09-09

**Authors:** Thomas Pröschold, Tatyana Darienko

**Affiliations:** 1 Research Department for Limnology, Leopold-Franzens-University of Innsbruck, Mondsee, Mondseestr. 9, A-5310 Mondsee, Austria; 2 Albrecht-von-Haller-Institute of Plant Sciences, Experimental Phycology and Culture Collection of Algae, Georg-August-University of Göttingen, D-37073 Göttingen, Germany

**Keywords:** *Choricystis*, *Lewiniosphaera*, Freshwater sponges, Endosymbiosis

## Abstract

Associations of freshwater sponges with coccoid green algae have been known for a long time. Two types of coccoid green algae, which are commonly assigned as zoochlorellae, are recognized by morphology: small coccoids (< 3 μm) without pyrenoids and larger *Chlorella*-like algae (4–6 μm) with pyrenoids. Despite their wide distribution in some freshwater sponges, these green algae were never studied using a combined analysis of morphology and molecular phylogeny. We investigated several endosymbiotic strains isolated from different *Spongilla* species, which were available in culture collections. Phylogenetic analyses of SSU and ITS rDNA sequences revealed that the strain SAG 211-40a is a member of the Chlorellaceae and represents a new species of the newly erected genus *Lewiniosphaera*, *L symbiontica*. The phylogenetic position was confirmed by morphology and ITS-2 barcode. The endosymbionts without pyrenoid were identified as *Choricystis parasitica* by morphology and phylogenetic analyses. The comparison with free-living strains revealed the recognition of two new *Choricystis* species, *C. krienitzii* and *C. limnetica*, which were confirmed by molecular signatures in V9 region of SSU rDNA and ITS-2 barcode.

**Electronic supplementary material:**

The online version of this article (10.1007/s13199-020-00711-x) contains supplementary material, which is available to authorized users.

## Introduction

Marine and freshwater sponges (Porifera) commonly contain photosynthetic endosymbionts. Connected with the fact that the endosymbionts need light for photosynthesis, they are only located in animals with colorless tissues or in organs and organelles exposed to sunlight. Most of marine sponges possess endosymbiotic cyanobacteria (coccoid or filamentous; Wilkinson 1992). Eukaryotic algae are less distributed in marine sponges. So far known the following eukaryotes have been recorded: *Symbiodinium* (Sara & Liaci 1964), cryptomonads (Wilkinson 1992), diatoms (Cox & Larkum 1983), coccoid red algae (Lemloh et al. 2009) and eustigmatophytes (Frost et al. 1997). In contrast, mostly coccoid green algae are known to live in symbiosis with freshwater sponges. Brandt (1882) was the first who recognized symbiotic associations between freshwater sponges and coccoid green algae. According to Manconi & Pronzato (2008), 219 species of freshwater sponges belonging to 45 genera were described. Zoochlorellae were only reported in ten genera (Penney & Racek 1968, Manconi & Pronzato 2002): *Spongilla*, *Eunapius*, *Radiospongilla*, *Ephydatia*, *Heteromeyenia*, *Anheteromeyenia*, *Umborotula*, *Corvomeyenia*, *Lubomirskia* and *Baikalospongia*. Unfortunately, details about the zoochlorellae are missing in the reports. Some morphological characterization about endosymbionts were only reported from *Spongilla lacustris* and *Spongilla fluviatilis* (Castro-Rodrigues 1930; Gilbert & Allen 1973; Reisser 1992 and references therein), *Radiospongilla sendai* (Handa et al. 2006), *R. cerebellata* (Handa et al. 2006), *Heteromeyenia stepanowii* (Handa et al. 2006), *Lubomirskia baicalensis* (Bil et al. 1999) and *Ephydatia fluviatilis* (Wilkinson 1980). Interestingly, several studies have shown that some freshwater and marine sponges can contain several different endosymbionts (Lemloh et al. 2009; Handa et al. 2006). Handa et al. (2006) provided the evaluation of endosymbionts of several Japanese freshwater sponges and postulated that they contain the representatives of genus *Choricystis*. Interestingly, one individual of *Radiospongilla cerebellata* contained two different coccoid algae – one clearly belonging to the genus *Choricystis* and second – *Chlorella*-like organism, possessed cup-shaped chloroplast with a single pyrenoid. Unfortunately, more detailed investigation of *Chlorella*-like algae was not conducted by Handa et al. (2006).

About the role of the green algal endosymbionts in freshwater sponges very little is known. Frost & Williamson (1980) demonstrated that *Spongilla lacustris* grew in aposymbotic form (without zoochlorellae) only up to 49% compared to the normal form with green algal symbionts. Cook (1983) found that the products of photosynthesis of zoochlorellae in sponges differed in their composition compared to *Hydra*-green algal association. In *Ephydatia fluviatilis*, glucose was the primary sugar, which were transferred to the host. In contrast, maltose was reported as primary carbohydrate, which was segregated to the *Hydra* host. Cook (1983) also found that the symbionts of *Ephydatia fluviatilis*, only transferred 9–17% of the total fixed carbon (in contrast to 25–30% in *Hydra*). This indicated that the mutual profit of both partners was the protection of the algae inside the sponge and the receiving of products (i.e., glucose and maltose) of algae photosynthesis by the sponge (Saller 1991). However, if all green algal endosymbionts found in freshwater sponges have the role in this association remained unresolved and need further studies. For those studies, it is of great importance to investigated both partners, which includes a clear identification of them at species level.

During our search for endosymbiotic *Chlorella*-like organisms in culture collections, we discovered at the SAG culture collection two strains (SAG 211-40a and SAG 211-40b) isolated by R. Lewin in 1956 from North American freshwater sponges *Spongilla lacustris* and *S. fluviatilis* (now *Ephydatia fluviatilis*) designated as *Chlorella* sp. Originally, the three strains designated as *Chlorella* sp. were isolated from different *Spongilla* individuals and different localities. One of these strains SAG 211-40c was taxonomically studied (Reisser & Widowski 1992; Pröschold et al. 2011). Kessler (1972) had detected maximal growth range for the strains SAG 211-40a and SAG 211-40c at 38 °C. Therefore, he designated both strains as *Chlorella vulgaris var. vulgaris* f. *tertia* (later as *Chlorella* III sensu Kessler, or *Chlorella sorokiniana*). SAG 211-40a and 211-40c can also survive at 2% NaCL (Kessler 1974, 1982), and a pH range from 3.0–6.0 (Kessler et al. 1991). Kessler (1976) assumed that both strains were identical belonging to *Chlorella sorokiniana* based on GC-content (78.6%) and similar physiological pattern. Pröschold et al. (2011) demonstrated that SAG 211-40c is representative of genus *Choricystis* based on morphology and SSU-ITS sequences. The aim of this study was to utilize an integrated morphological and molecular approach to characterize the remaining two strains isolated by Lewin (SAG 211-40a and SAG 211-40b).

## Material and methods

The two strains SAG 211-40a and SAG 211-40b were originally isolated by Ralph A. Lewin from two *Spongilla* specimen collected from two sites in Massachusetts (USA) in 1956. SAG 211-40a and SAG 211-40b were the endosymbionts of *Spongilla lacustris* and *S. fluviatilis* (= *Ephydatia fluviatilis*) found in Cotuit and Manumet Beach (Lewin 1966). Both strains were cultivated on agarized Bold’s basal medium (3N-BBM+V; medium 26a in Schlösser 1997). For morphological investigations, we studied three additional strains for comparisons. The strains SAG 251–1, SAG 251–2 and SAG 17.98 assigned as *Choricystis* originated from different freshwater habitats and are free-living. They were also cultivated under the standard conditions (18 °C, with 50 μmol photons/m^2^s^1^ provided by daylight fluorescent tubes, Osram L36W/954 Lumilux de lux daylight, Munich, Germany, and light:dark cycle of 16:8 h for two to three weeks). The light microscopic investigations were conducted using an Olympus BX-60 microscope (Olympus, Tokyo, Japan) and the micrographs were taken with a ProgRes C14plus camera using the ProgRes CapturePro imaging system (version 2.9.0.1), both from Jenoptik, Jena, Germany).

The genomic DNA of the strains were extracted using the DNeasy Plant Mini Kit (Qiagen, Hilden, Germany) following the instructions provided by the manufacturer. The SSU and ITS rDNA was amplified in PCR reactions using the Taq PCR MasterMix Kit (Qiagen, Hilden, Germany) with the primers EAF3 and ITS055R (Marin et al. 2003). The PCR product was purified and sequenced as described by Darienko et al. (2019). The SSU and ITS rDNA sequence is available in the EMBL, GenBank and DDBJ sequence databases under the accession number given in the figures. The sequences were aligned and separated into two data sets: (i) The SSU and ITS rDNA sequence of strain SAG 211-40a was included in a data set of a total of 41 sequences (2602 bp) of representatives of the Chlorellaceae (Trebouxiophyceae). (ii) For the other data set, the SSU rDNA sequences of the other strains were checked about their phylogenetic positions using BLAST N search algorithm (Altschul et al. 1990) to find out their generic affiliation. All resulting SSU rDNA sequences of a minimal length of 1500 bp were included in a preliminary data set. Representatives of each phylogenetic lineage were put into a reduced SSU data set (13 taxa, 1780 bp) for the detailed analyses. The data sets were aligned according to the secondary structures.

For the phylogenetic analyses, the datasets with unambiguously aligned base positions were used. To test which evolutionary model was the best fit for both data sets, we calculated the log-likelihood values of 56 models using the automated model selection tool implemented in PAUP, version 4.0b167 (Swofford 2002), and the best model according to the Akaike criterion by PAUP were chosen for the analyses. The settings of the best models are given in the figure legends. The following methods were used for the phylogenetic analyses: distance, maximum parsimony, maximum likelihood, and Bayesian inference. Programs used included PAUP version 4.0b167 (Swofford 2002), RAxML version 8.2.12 (Stamatakis 2014), MrBayes version 3.2.7a (Ronquist et al. 2012) and the PHASE package 2.0 (Jow et al. 2002, Higgs et al. 2003, Hudelot et al. 2003, Gibson et al. 2005, Telford et al. 2005). For the Bayesian calculations, the secondary structure models of SSU and ITS (doublet in MrBayes and RNA7D in PHASE) have been taken.

The secondary structures of ITS-2 sequences were folded using the computer programs mfold (Zuker, 2003), which used the thermodynamic model (minimal energy). The following three constraints were set for the folding: (1) the last 25 bases of the 5.8S rRNA and the first 25 of the LSU rRNA must bind and form the 5.8S/LSU stem, (2) the pyrimidine/pyrimidine mismatch (the first RNA processing site) after the 5-7th base pair in Helix II must be present in the structure, and (3) the second RNA processing site, the GGU motif characteristic for green algae, must be at the 5’ site in Helix III (for details about the processing sites and constraints; see Coleman 2003, Cote et al. 2002).

The secondary structure models of ITS-2 derived from these folding results were then used for species delimitation within *Choricystis*. For the ITS-2/CBC approach, the conserved region of ITS-2 was extracted following the procedure that was introduced for *Coccomyxa* by Darienko et al. (2015): it includes (1) 16 base pairs of the 5.8S/LSU stem, (2) five base pairs of Helix I, (3) eleven base pairs of Helix II including the pyrimidine-pyrimidine mismatch, and (4) all base pairs of Helix III. The resulting data set was then manually aligned. These alignments have been translated into base pair alignment by using a number code for each base pair (**1** = A-U; **2** = U-A; **3** = G-C; **4** = C-G; **5** = G•U; **6** = U•G; **7** = mismatch; **8** = deletion/insertion or single bases). The barcodes for each species were compared to detect for compensatory base changes (CBCs), hemi-CBCs (HCBCs), insertions/deletions, and single or unpaired bases. The secondary structures were visualized using the programs PseudoViewer (Byun & Han 2009) and VARNA (Darty et al. 2009).

To get an overview about the distribution of the investigated strains, we used the variable regions of the SSU (V4 and V9) as well as the ITS-2 for discovery of haplotypes belonging to each lineage. This was necessary because almost no complete SSU and ITS rDNA sequences were available in GenBank. The BLAST N search results (100% coverage, >97% identity; Altschul et al., 1990) are summarized in Table S1. To construct the haplotype networks of each region, we used the TCS network tool (Clement et al. 2000, 2002) implemented in PopART (Leigh & Bryant 2015) for *Choricystis*.

## Results

### Morphology and phenotypic plasticity of the green algal endosymbionts of freshwater sponges

**Fig. 1:**
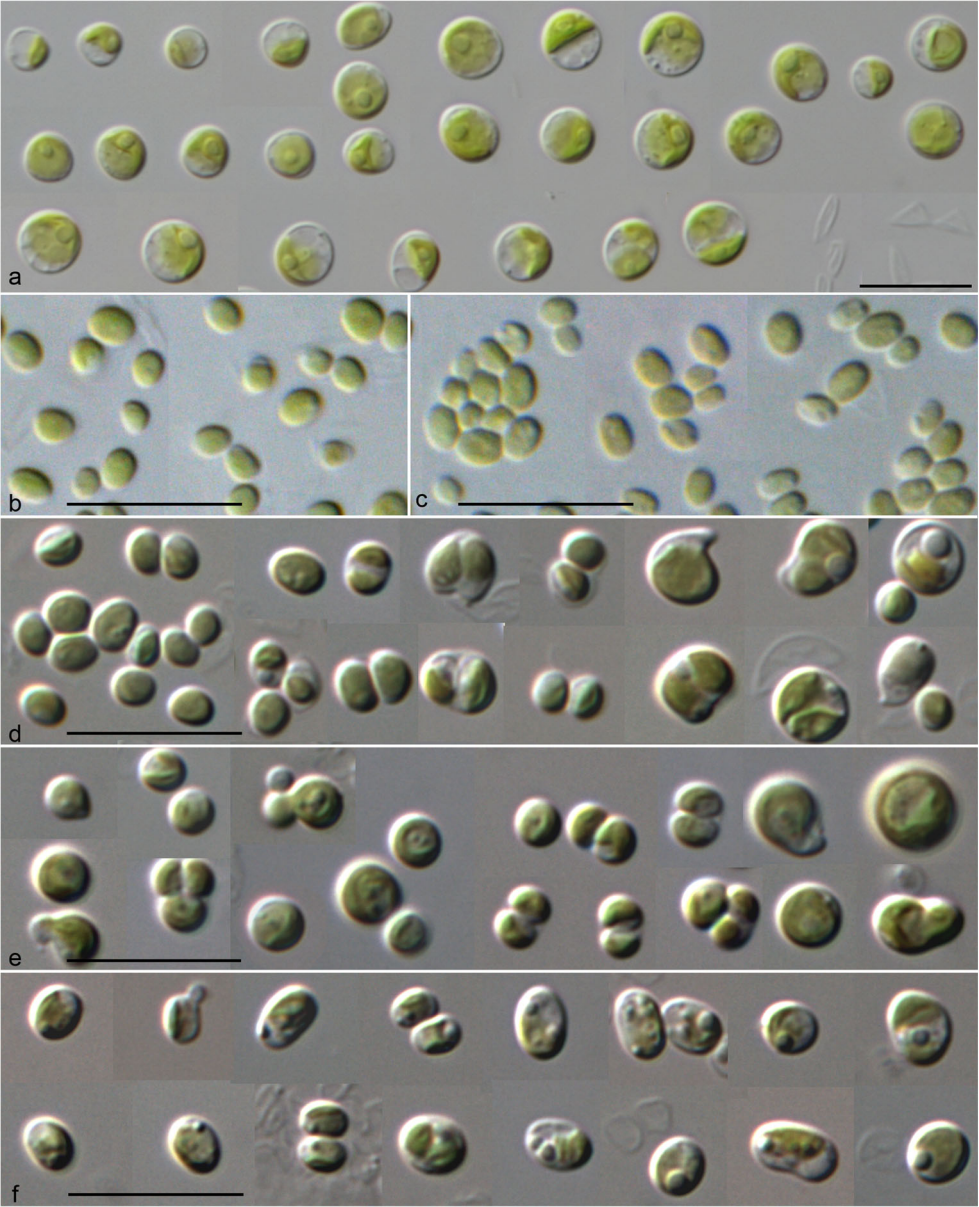
Morphology and phenotypic plasticity of the investigated strains. **a.** *Lewiniosphaera symbionica* gen. et sp. nov., strain SAG 211-40a; **b.** *Choricystis parasitica*, strain SAG 211-40b; **c.** *C. parasitica*, strain SAG 211-40c; **d.** *C. parasitica*, strain SAG 251–1; **e.** *C. limnetica* sp. nov., strain SAG 251–2; **f.** *C. krienitzii* sp. nov., strain SAG 17.98; scale bar = 10 μm

The investigated strains were not identical in morphology. The strain SAG 211-40a differed from all other strains by a larger cell shape (ellipsoid-spherical) and size (3.5–8.0 μm) and cup-shaped chloroplast containing a single pyrenoid (Fig. 1a). This strain showed similarity to *Chlorella vulgaris*/*C. sorokiniana* and could be identified as this species if the identification key of Komarek & Fott (1983) is used. In contrast, all other strains differed by smaller cell sizes (2–4 μm) and ovoid to ellipsoidal, sometimes almost spherical cell shapes (Fig. 1b-f). Older cells sometimes formed irregular or bigger spherical shapes. The cells had single parietal chloroplasts without pyrenoids. Using the identification key, all strains were identified as *Choricystis minor* (= *C. parasitica* according to Pröschold et al. 2011). The strains isolated from sponges (SAG 211-40b and SAG 211-40c) showed only small morphological differences compared to the other free-living strains SAG 251–1, SAG 251–2, and SAG 17.98 (Fig. 1b-f).

### Molecular phylogeny of the sponge endosymbionts

**Fig. 2:**
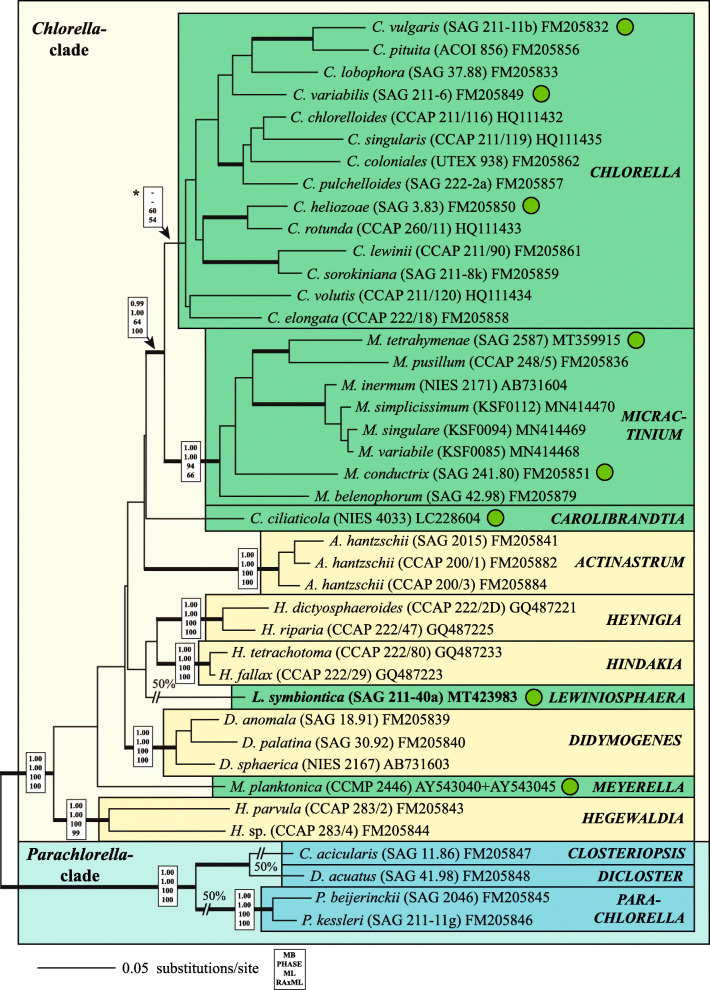
Molecular phylogeny of the Chlorellaceae based on SSU and ITS sequence comparisons. The phylogenetic tree shown was inferred using the maximum likelihood method based on the data set (2602 aligned positions of 41 taxa) using PAUP 4.0a167. For the analyses the best model was calculated by PAUP. The setting of the best model was given as follows: GTR + I + G (base frequencies: A 0.2096, C 0.2853, G 0.2641, T 0.2410; rate matrix A-C 0.6832, A-G 1.0225, A-U 0.8708, C-G 0.6148, C-U 2.7613, G-U 1.0000) with the proportion of invariable sites (I = 0.6819) and gamma shape parameter (G = 0.4812). The branches in bold are highly supported in all analyses (Bayesian values >0.95 calculated with MrBayes and PHASE, 10 million generations; bootstrap values >50%, calculated with PAUP, 1000 replicates using maximum likelihood, neighbor-joining, and maximum parsimony). The endosymbiotic species are marked with a green circle. Genera containing endosymbiotic species are highlighted in green boxes. The accession and strain numbers are given after the species names

**Fig. 3:**
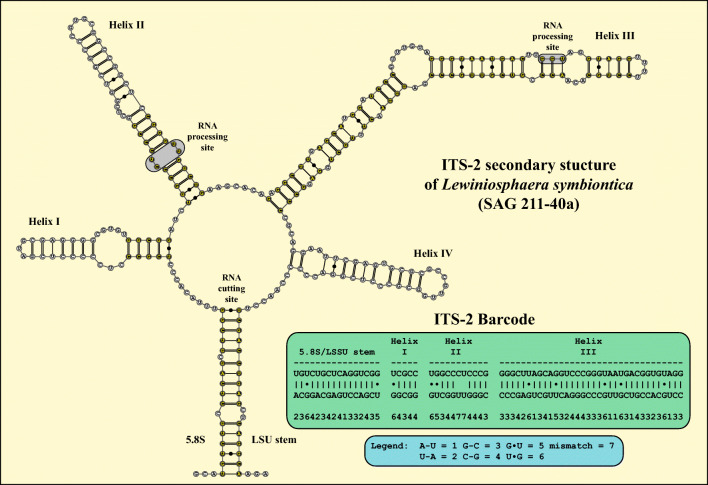
ITS-2 secondary structure of *Lewiniosphaera symbionica* gen. et sp. nov., strain SAG 211-40a. The base pairs used for the ITS-2 barcode are highlighted in black. The ITS-2 barcode is given as number-code

To discover to which evolutionary lineage the investigated strains belong, the BLAST N search of the SSU and ITS rDNA sequences were applied. The results showed that the strain SAG 211-40a is a member of the Chlorellaceae (Fig. 2) and the other strains belong to the genus *Choricystis*. For the phylogenetic analyses, two approaches were used depending on the availability of reference sequences in GenBank. For the Chlorellaceae, a data set of complete SSU and ITS rDNA sequences for all representative species was available. Therefore, the strain SAG 211-40a was included in this data set and the phylogenetic analyses using complex evolutionary models revealed that this strain represents an own lineage within the *Chlorella* clade. The SSU sequence of SAG 211-40a contains two group I introns at positions 323 and 1046 (in *E. coli* reference), which is absolutely identical to the GenBank entry X73993 (designated as *Chlorella sorokiniana* in Huss et al.*,* 1999). The ITS-2 secondary structure and the ITS-2 barcode (Fig. 3) showed that this strain represents a new species, which is described below.

**Fig. 4:**
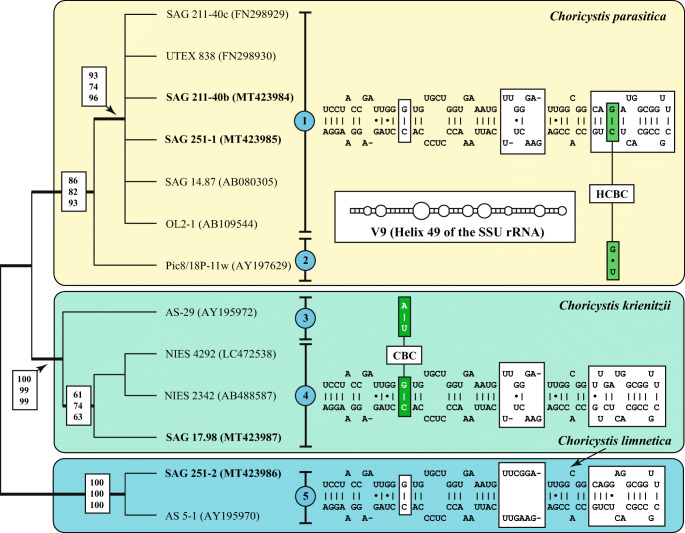
Molecular phylogeny of the genus *Choricystis* based on SSU sequence comparisons. The phylogenetic tree shown was inferred using the maximum likelihood method based on the data sets (1780 aligned positions of 13 taxa) using PAUP 4.0a167. For the analyses the best model was calculated by PAUP. The setting of the best model was given as follows: TrN + I (base frequencies: A 0.2485, C 0.2140, G 0.2773, T 0.2602; rate matrix A-C 1.0000, A-G 2.5538, A-U 1.0000, C-G 1.0000, C-U 10.1959, G-U 1.0000) with the proportion of invariable sites (I = 0.9235). The branches in bold are highly supported in all analyses (bootstrap values >50%, calculated with PAUP, 1000 replicates using maximum likelihood, neighbor-joining, and maximum parsimony). The accession and strain numbers are given after the branches. The variants of the V9 haplotypes are marked with a blue circle. The V9 secondary structures of the SSU rDNA for each species color-coded are given in boxes. The variable regions in the V9 are highlighted in white boxes. Compensatory base changes (CBCs and HCBCs) are marked in green boxes

**Fig. 5:**
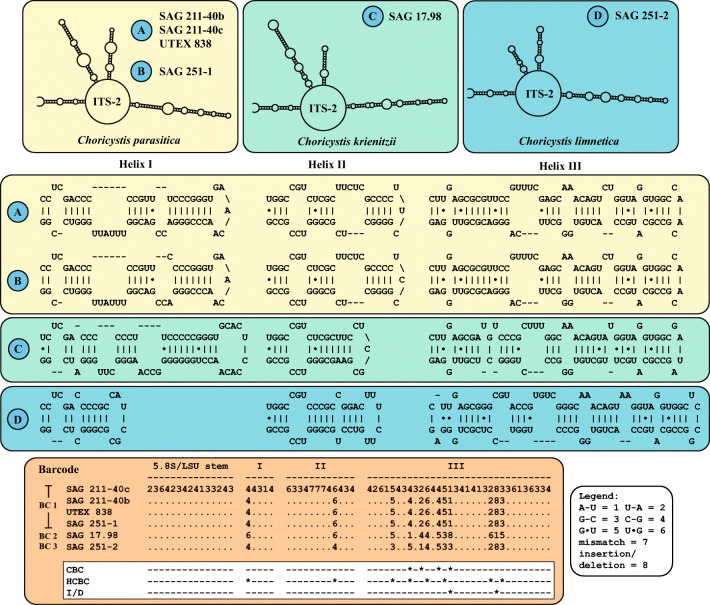
ITS-2 secondary structures of the three *Choricystis* species. The helices I-III are given for each species and the conserved regions have been taken for the ITS-2 barcodes, which are summarized as number-codes. Compensatory base changes (CBCs and HCBCs) and insertion/deletion are marked with asterisks

For the phylogenetic analyses of the four remaining strains (SAG 211-40b, SAG 251–1, SAG 251–2 and SAG 17.98), it was necessary to use a different approach because only two SSU and ITS rDNA sequences (SAG 211-40c and UTEX 838) were available in GenBank. At first, the introns of the SSU rDNA sequences were excluded. Whereas the strain SAG 211-40b did not contain an intron in its SSU, the other strains had one or up to four introns (SAG 251–1 at positions 1046 and 1512, SAG 251–2 at position 1046, and SAG 17.98 at positions 323, 943, 1046 and 1512 according to the *E. coli* reference). Then, we searched for SSU rDNA sequences longer than 1500 bp in GenBank using the BLAST N search (see Material & Methods) and did preliminary analyses to find the variable entries. 24 SSU rDNA sequences representing four additional lineages were discovered. Representatives of these lineages were analyzed together with newly sequenced strains. As demonstrated in Fig. 4, three clades could be revealed in the maximum likelihood analysis, which are highly supported in the bootstrap analyses. Among *Choricystis*, 49 out of 1780 bp were variable (2.8%). Many of the variable positions were located in the V9 region of the SSU. Comparing the V9 secondary structures five characteristic haplotypes (1–5) could be revealed (Fig. 4). The haplotypes 1 and 2 differed only by one HCBC and 3 and 4 by one CBC from each other (highlighted in green boxes in Fig. 4). To discover if these haplotypes represent biological species, the ITS-2 secondary structures was investigated using the ITS-2/CBC approach introduced by Darienko et al. (2015). Unfortunately, no ITS rDNA sequences of the haplotypes 2 and 3 were available in GenBank. The remaining haplotypes represent three species, *Choricystis parasitica* and two new species (*C. krienitzii* and *C. limnetica*), which are described below. The ITS-2 secondary structures of the three species showed only three helices I-III. Helix IV is missing (Fig. 5). All three helices varied among the three species. The three strains isolated from freshwater sponges (A in Fig. 5) differed slightly (two bases in variable regions) from the free-living strain SAG 251–1 (B in Fig. 5), but still represent to the same species. The separation into three species is supported by four CBCs and eight HCBCs in the conserved region of ITS-2.

### Distribution of the sponge endosymbionts

**Fig. 6:**
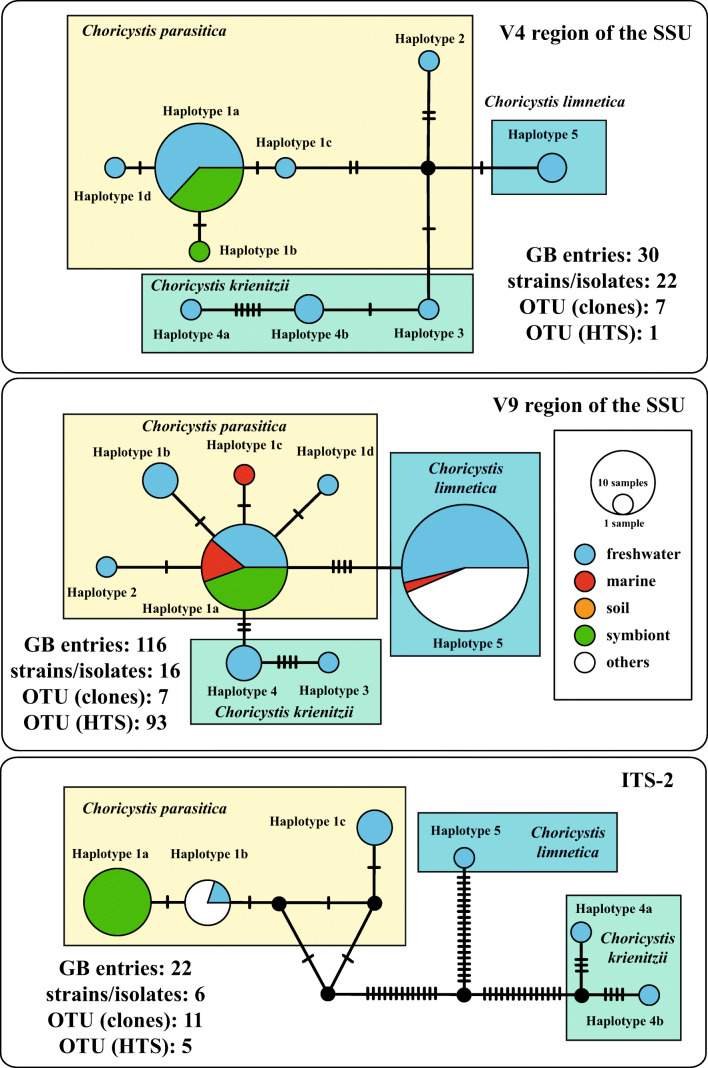
TCS haplotype network inferred from V4 and V9 of the SSU rDNA as well as ITS-2 rDNA sequences of the three *Choricystis* species. This network was inferred using the algorithm described by Clement et al. (2002). Sequence nodes corresponding to samples collected from different habitats

**Fig. 7:**
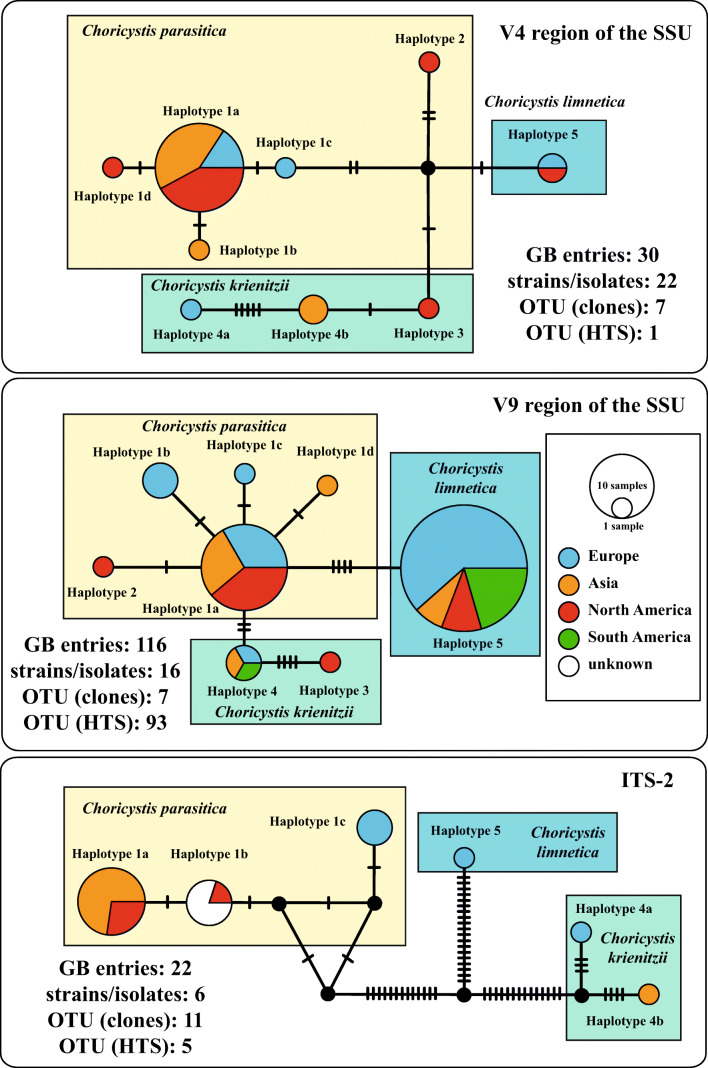
TCS haplotype network inferred from V4 and V9 of the SSU rDNA as well as ITS-2 rDNA sequences of the three *Choricystis* species. This network was inferred using the algorithm described by Clement et al. (2002). Sequence nodes corresponding to samples collected from different geographical regions

To get a better overview about specificity and geographical distribution of the species discovered in our study, we checked the variable regions V4 and V9 commonly used for species detection in environmental studies as well as the ITS-2. The BLAST N search results of these regions were summarized in Table S1. For each region, the haplotypes were discovered and named according to numbers used in Fig. 4 (variations are highlighted with lowercase letters). For SAG 211-40a, only two entries (MK248022 and KM514836) using ITS-2 could be found in GenBank (V4 and V9 cannot distinguish among the genera belonging to the *Chlorella* clade). For the three species of *Choricystis*, 30, 116 and 22 GenBank entries were discovered using the three regions V4, V9 and ITS-2, respectively. To create haplotype networks, we collected the metadata of the entries (habitat and geographical origin) and used the TCS approach implemented in PoPART for graphical presentation. As demonstrated in Fig. 6, all endosymbiotic *Choricystis* belonged to haplotypes 1a or 1b of *C. parasitica*. The free-living (freshwater and soil) entries were distributed among the three species. In contrast, marine records could only be found in *C. parasitica* and *C. limnetica*. The three species were distributed in Europe, Asia and North America (Fig. 7) without any tendencies of endemism. No evidence of the presence of *Choricystis* in the other regions around world has been published yet. HTS data have shown that *C. limnetica* is also present in South America, which indicates that the current distribution pattern is based on too few studies.

## Discussion

### Multiple origin of symbionts in freshwater sponges

Brandt (1882) discovered the endosymbiosis between freshwater sponges and green coccoid algae. Since then sponge-algal associations became one of most prominent systems to study endosymbiosis. The differentiation between symbiotic and free-living algae is very difficult because sponges filter water and it cannot be easy distinguished if the algae are symbionts or only food. To avoid misinterpretations, only gemmules and archeocytes were usually taken for investigation of endosymbionts in sponges (Williamson 1979, Masuda 1985, 1990; Handa et al. 2006, Chernogor et al. 2013). Van Trigt (1919) and van Weel (1949) identified endosymbionts of freshwater sponges as members of polyphyletic genus *Pleurococcus*. Lewin (1966) isolated several strains of endosymbionts of *Spongilla lacustris* and designated them as unidentified species of *Chlorella*. Despite the cell size of 2–3 μm, no further information about morphology was given. Huss et al. (1986) identified three isolates of Lewin (SAG 211-40a, SAG 211-40b and SAG 211-40c) as *Chlorella sorokiniana* based on DNA hybridization. The SSU rDNA of the first strain was sequenced confirming the close relationship to *C. sorokiniana* (Huss et al. 1999). Our study revealed that this strain represents a new species (*Lewiniosphaera symbiontica*; see below). Pröschold et al. (2011) have demonstrated that the strain SAG 211-40c belonged to *Choricystis parasitica*, which is identical in morphology and SSU and ITS rDNA sequences with strain SAG 211-40b (Figs. 1, 4-5).

Masuda (1990) investigated gemmules of different species of freshwater sponges and detected that the large part of the investigated species contained coccoid pyrenoid-less green algae and only *Radiospongilla sendai* had two organisms, one coccoid without pyrenoid and one with pyrenoid. Handa et al. (2006) re-investigated four species of freshwater sponges including *Radiospongilla cerebellata* that also contained two different endosymbionts with and without pyrenoid. Isolated cultures were morphologically studied but unfortunately no strain was deposited to culture collections so that no further studies are possible. Comparing these morphological studies with our results, it seems that freshwater sponges have mostly *Choricystis parasitica* and only occasionally *Chlorella*-like algae as endosymbionts. If the *Chlorella*-like algae in sponges are always *Lewiniosphaera symbiontica*, this cannot be decided at present and need further investigations. The ITS-2 BLAST N search of this species only revealed two entries in GenBank (MK248022 and KM514836), both free-living in Chinese freshwater habitats according to the metadata provided.

Different endosymbionts have been found in endemic freshwater sponges from Lake Baikal (Russia). Annenkova et al. (2011) reported that *Baikalospongia intermedia*, *B. recta* and *Lubomirskia incrustans* were exclusively associated with dinoflagellates. In contrast, Kulakova et al. (2014) provided partial *rbc*L sequences of species belonging to the Lubomirskiidae that showed high similarity to those of *Choricystis parasitica*. Unfortunately, the published sequences were too short for a precise species identification. Chernogor et al. (2013) described *Mychonastes jurisii*, a coccoid green alga which belongs to the Chlorophyceae, as endosymbiont of *Lubomirskia baicaliensis*. It seems that the endosymbionts of freshwater sponges found in Lake Baikal are not species specific. However, Kulakova et al. (2014) demonstrated that *Baikalospongia bacillifera* and *Lubomirskia incrustans* had the same *Choricystis parasitica*-like organism, which were confirmed in our study. *Choricystis parasitica* was also reported from two specimens of unidentified *Ephydatia* species by Zhu et al. (2016), which were confirmed by our study (see Table S1). The three lineages representing species as demonstrated in our study were also found in the phylogenies using the chloroplast *rbc*L and 16S (Kulakova et al. 2020). All Choricystis endosymbionts found in freshwater sponges belonged to *C. parasitica.* Only *Baikalospongia intermedia* and *Lubomirskia abietina* seemed to have *C. krienitzii* as endosymbionts (Kulakova et al. 2020). However, this needs further studies and confirmation by sequencing of ITS, the commonly used marker for species delimitation in green algae.

### Distribution patterns of sponge endosymbionts

As described above, some freshwater sponges with green algae as endosymbionts have a worldwide distribution. However, very little is known about the identity of these green algae and their distribution. The two morphological types were discovered, the *Chlorella*-like and *Choricystis*-like coccoid green algae. As shown in Figs. 1 and 3, the first one represents a new lineage within the *Chlorella* clade (*Lewiniosphaera* gen. nov.; see below), and the second *Choricystis parasitica*. *Lewiniosphaera* was reported free-living twice in China (MK248022 and KM514836), which could be only identified using the ITS-2 BLAST N search. The V4 and V9 regions of *Lewiniosphaera* is not diagnostic and can therefore not be detected in HTS approaches. In contrast, these regions as well as ITS-2 could identify *Chloricystis* at species level. Therefore, we checked the distribution pattern of the three species using these regions. So far known, the three *Choricystis* species occurred in freshwater habitats free-living or as endosymbiont in sponges or *Paramecium bursaria* (see Nakahara et al., 2004) as well as in soil and rarely in marine environments. Interestingly, endosymbiotic *Choricystis* represented only in the haplotypes 1a and 1b of *C. parasitica* (Fig. 6). However, the distribution pattern could be biased for the following reasons: The results using V4 and ITS-2 mainly based on sequencing of isolated strains or clones, whereas the V9 entries mostly came from HTS approaches. Our study showed that the strains contained introns at different positions. Especially introns at position 1512 could lead in an underrepresentation of *Choricystis* species in HTS studies using PCR amplicons. The often used reverse primers such as BR often did not bind if this intron is present. This could explain why *C. limnetica* have been found in many freshwater bodies, whereas only few records were reported using V9 for the other two species because both often had the intron 1512. Similar is the situation for the V4 region of the SSU. All three species had either introns before and/or after the V4 regions. Therefore, we think that *Choricystis* in freshwater habitats is generally wider distributed, but they were probably not detected in HTS studies because of the highlighted PCR bias. Fawley et al. (2005) showed a high diversity of these picoplanktonic organisms using culture-dependent approach. They demonstrated that *Choricystis* is widely distributed in North American lakes. Krienitz et al. (1996) found *Choricystis minor* (now *C. parasitica*) in two lakes Northeast Germany. Many studies showed that *Choricystis* is widely distributed (Hindak 1980, 1984, Komarek & Fott 1983, Kulakova et al. 2020). However, they were probably often overlooked in monitoring programs of investigation for water quality because of its cell size (<3 μm). In contrast, Metz et al. (2019) mostly found *C. limnetica* in freshwater and soil samples collected around the world. Both other species were not reported in that study, which could be the result of the intron distribution described above.

The problems in PCR amplification mentioned could be probably resolved by using PCR-independent approach such as Oxford Nanopore sequencing, but has not been proven yet. Therefore, the geographical distribution pattern presented in Fig. 7 should be only taken as preliminary indications of the distribution of *Choricystis*.

### Taxonomic consequences and remarks to the genus*Choricystis*

The investigated strains belong to two groups among the Trebouxiophyceae, the *Chlorella* clade (Fig. 2) and the genus *Choricystis* (Fig. 4). The comparison of SAG 211-40a with the described species belonging the Chlorellaceae have revealed that this strain represents a new genus and species, which is described as follows:

***Lewiniosphaera*** Pröschold & Darienko, gen. nov.

**Description**: Young cells are solitary, broadly ellipsoidal, slightly polygonal or irregular; 3.5–4.5 μm. Mature vegetative cells are broadly ellipsoidal, ovoid, spherical, up to 5.0 × 5.5 μm in size. Old cells are spherical up to 8.0 μm in diameter. Chloroplast parietal cup-shaped possesses a single pyrenoid surrounded by starch grains. Cytoplasm is vacuolized. Asexual reproduction by autosporulation. The 2–4 autospores are produced per cell. Autosporangia are 5.0 × 6.5 μm in size. Autospores are released after rupture of the mother cell wall.

**Diagnosis**: Differs from other genera by its SSU sequence.

**Etymology**: The genus was named in honor of Prof. Dr. Ralph A. Lewin (1921–2008) for his contributions to phycology.

**Type species**: ***Lewiniosphaera symbiontica*** Pröschold & Darienko, sp. nov.

***Lewiniosphaera symbiontica*** Pröschold & Darienko, sp. nov. (Fig. 1a).

**Description**: with the features of the genus.

**Diagnosis**: Differs from morphologically similar *Chlorella*-like algae by genetic signatures in SSU and ITS-2 rDNA sequences (GenBank: MT423983) as well as in ITS-2 Barcode (Fig. 3).

**Holotype** (designated here): The authentic strain SAG 211-40a cryopreserved in metabolically inactive state at SAG under the number Z000693530.

**Type locality**: *Spongilla lacustris*, endosymbiont collected at Cotuit, Barnstable County, Massachusetts, USA.

The other investigated strains belong to *Choricystis*, which was proposed by Skuja (1948) for the section of the genus *Coccomyxa* (*Eucoccomyxa* and *Choricystis*) for unicellular *Coccomyxa*-like algae without distinct mucilage. He pointed out that *Choricystis* species are morphological very similar to *Nannochloris* Naumann (1919) and some small *Stichococcus*-like species. Within this section, Skuja described two new species, *Coccomyxa minor* and *C. coccoides*. Fott (1976) changed the status of Skuja’s section, described it as new genus *Choricystis* and transferred both species into it. In addition, he described two other species *C. granulata* (now *Siderocelis granulata*) and *C. chodatii* (now *Coccomyxa chodatii*). Currently eight morphological species of *Choricystis* are recognized. Unfortunately, for most species only figures were presented as holotype and no reference strains are available in public culture collections. Only *C. parasitica* was revised by Pröschold et al. (2011). Komarek & Fott (1983) subdivided the genus into two morphological groups according to their sizes, one with cells over 4–6 μm long and other group up to 4.0 μm. Krienitz et al. (1996) mentioned that true *Choricystis* species are picoplanktonic organisms with cell size up to 3.0–4.0 μm. Take it into account, only three species of *Choricystis* could be recognized based on cell size and shape. *Choricystis parasitica* (= *C. minor*), *C. coccoides* (with a cell size of 1.5 μm) and *C. hindakii*. Krienitz et al. (1996) demonstrated that *C. minor* (*= C.parasitica*) and *C. hindakii* represented the same species. However, a formal proposal has not been published yet. Species with a cell size over 4.0–6.0 μm are not members of *Choricystis* and should be excluded from this genus.

The investigated *Choricystis* strains were morphologically very similar and could be identified as *C. minor*, which is a synonym of *C. parasitica* as described in Pröschold et al. (2011). Surprisingly, the strains SAG 251–2 and SAG 17.98 were genetically different compared to those belonging to *C. parasitica*. They represent two further species of the genus *Choricystis*. The strain SAG 251–2 differed slightly by the spherical cell shape from the other isolates. The spherical cell shape is typical for *C. coccoides*, but SAG 251–2 differed by bigger cell size. As consequence, we propose the following taxonomical changes and two new species for the strains SAG 251–2 and SAG 17.98:

***Choricystis parasitica*** (K. Brandt) Pröschold & Darienko (Fig. 1b-d).

**Basionym**: *Z. parasitica* K. Brandt, 1881a, Verh. Physiol. Ges. Berlin 4, p. 24.

**Synonyms**: *Coccomyxa minor* Skuja, 1948, Taxonomie des Phytoplanktons, Symb. Bot. Upsaliensis 9 (3), p. 146, Table XVI, Fig. 23; *Choricystis minor* (Skuja) Fott (1976), Algol. Stud. 17, p. 384; *Choricystis hindakii* Tell, 1979, Schweiz. Z. Hydrolog. 41: 152, fig. 1.

**Emended diagnosis**: SSU and ITS sequences (GenBank: FN298929) and ITS-2 Barcode: BC1 in Fig. 5.

**Comment**: The strains SAG 211-40b (Fig. 1b) and SAG 211-40c (Fig. 1c) isolated from the freshwater sponges *Spongilla lacustris* and *S. fluviatilis* were morphologically identical, the free-living strain SAG 251–1 (Fig. 1c) differed only by slightly bigger cell size. *Choricystis hindakii* described by Tell (1979) differed from *C. minor* only by bean-like cell shape, which is not stable in culture. Therefore, Krienitz et al. (1996) indicated that this species should be treated as synonym of *C. minor* (now *C. parasitica*).

***Choricystis krienitzii*** Pröschold & Darienko sp. nov. (Fig. 1f).

**Description**: Cells are solitary, oval or ovoid sometimes pyriform or spherical, without mucilage. Cell wall smooth without granules. Cell size 2.2 × 2.8 up to 3.2 × 4.2 μm. Single chloroplast is parietal and covers almost the whole cell. Chloroplast does not contain starch grains, but polyphosphate granules and lipids bodies. Pyrenoid is absent. One nucleus could be seen only on TEM. 2- rarely 4 autospores are formed during reproduction, which are released by rupture of the mother cell wall. Sometimes budding-like reproduction was observed.

**Diagnosis**: Differs from morphologically similar *Choricystis* species by genetic signatures in SSU and ITS sequences (GenBank: MT423987) and ITS-2 Barcode: BC2 in Fig. 5.

**Holotype** (designated here): The authentic strain SAG 17.98 cryopreserved in metabolically inactive state at SAG.

**Type locality**: Freshwater pond at the Old Botanical Garden of the University of Göttingen.

**Etymology**: The genus was named in honor of Dr. Lothar Krienitz for his contributions to phycology and limnology.

***Choricystis limnetica*** Pröschold & Darienko sp. nov. (Fig. 1e).

**Description**: Cells are solitary, broadly ellipsoidal or spherical, without mucilage, uninucleate. Cell wall smooth without granules. Cell size 2.3 × 1.7 up to 3.1 × 2.9 μm. Single chloroplast is parietal and covers almost the whole cell. Chloroplast without starch grains but contains polyphosphate granules and lipids bodies. Pyrenoid is absent. Reproduction by autospores formation of 2 rarely 4 autospores released by rupture of the mother cell wall. Sometimes budding-like reproduction was observed.

**Diagnosis**: Differs from morphologically similar *Choricystis* species by genetic signatures in SSU and ITS sequences (GenBank: MT423986) and ITS-2 Barcode: BC3 in Fig. 5.

**Holotype** (designated here): The authentic strain SAG 251–2 cryopreserved in metabolically inactive state at SAG.

**Type locality**: Water tank, Schlieren, Switzerland.

## Electronic supplementary material

Table S1 Haplotype designations of the gene regions (V4, V9 and ITS-2) and the grouping to geographical region and habitats (used for the TCS networks; see Figs. 6-7) for each *Choricystis* species. The records of *C. limnetica* reported in Metz et al. (2019) could not be linked to the geographical origin and habitat and were therefore marked with an asterisk. (PDF 188 kb)
