# Supplementary material for: Choricystis and Lewiniosphaera gen. nov. (Trebouxiophyceae Chlorophyta), two different green algal endosymbionts in freshwater sponges
Source: Symbiosis. 2020 Sep 9;82(3):175–88. doi: 10.1007/s13199-020-00711-x (PMC7725700; doi:10.1007/s13199-020-00711-x)
Supplement: Supplementary file 1 — Haplotype designations of the gene regions (V4, V9 and ITS-2) and the grouping to geographical region and habitats (used for the TCS networks; see Figs. 6-7) for each Choricystis species. The records of C. limnetica reported in Metz et al. (2019) could not be linked to the geographical origin and habitat and were therefore marked with an asterisk. (PDF 188 kb) [file 13199_2020_711_MOESM1_ESM.pdf]

Table S1. Haplotype designations of the gene regions (V4, V9 and ITS-2) and the grouping to geographical region and habitats (used for the TCS networks; see Figs. 6-7) for each *Choricystis* species. The records of *C. limnetica* reported in Metz et al. (2019) could not be linked to thegeographical origin and habitat and were therefore marked with an asterisk.

Haplotype	V4	strain/clone/OTU	habitat	region	origin
1a	FN298929	SAG 211-40c	symbiont	North America	USA: Massachusetts, Manumet Beach, endosymbiont from Spongilla lacustris
1a	FN298930	UTEX 838	symbiont	North America	USA, endosymbiont from Ephydatia fluviatilis
1a	MT423984	SAG 211-40b	symbiont	North America	USA: Massachusetts, Manumet Beach, endosymbiont from Ephydatia fluviatilis
1a	MT423985	SAG 251-1	freshwater	North America	Canada: Quebec, Lac Grandpré
1a	AY543052	Itas 9/21 S-1w	freshwater	North America	USA: Minnesota, Lake Itasca
1a	AY195975	Itas 9/21 14-5w	freshwater	North America	USA: Minnesota, Lake Itasca
1a	AY543051	Itas 9/21 14-10w	freshwater	North America	USA: Minnesota, Lake Itasca
1a	KX139550	isolate HB-2	symbiont	Asia	China: Hubei, Lake Ziyang, endosymbiont of Spongilla sp.
1a	KX139548	isolate TB-2	symbiont	Asia	China: Tibet, Nagtsang Tso, endosymbiont of Spongilla sp.
1a	KX139549	isolate HB-1	symbiont	Asia	China: Hubei, Lake Ziyang, endosymbiont of Spongilla sp.
1a	GU067789	ESS220206.010	freshwater	Europe	Luxembourg: Lake Esch sur Sure
1a	AB109544	OL2-1	symbiont	Asia	USA: Florida, Orlando, ditch, endosymbiont of Paramecium bursaria
1a	AY220082	MDL5-9	freshwater	North America	USA: North Dakota, Mud Lake, Arrowwood National Wildlife Refuge
1a	AH012392	1A1	freshwater	Europe	Switzerland: Lake Hagel
1a	AB488586	NIES-2338	freshwater	Asia	Japan: Lake Toya, Hokkaido
1a	AB488585	NIES-2337	freshwater	Asia	Japan: Lake Shikotsu, Hokkaido
1a	AB488584	NIES-2335	freshwater	Asia	Japan: Lake Kuttara, Hokkaido
1a	AH012393	3D4	freshwater	Europe	Switzerland: Lake Alpnach
1a	AB488792	NIES-2333	freshwater	Asia	Japan: Lake Misuzu, Nagano
1b	KX139547	isolate TB-1	symbiont	Asia	China: Tibet, Nagtsang Tso, endosymbiont of Spongilla sp.
1c	AB080305	SAG 14.87	freshwater	Europe	United Kingdom: River Crouch
1d	AY197623	MDL1/12-8	freshwater	North America	USA: North Dakota, Mud Lake, Arrowwood National Wildlife Refuge
2	AY197629	Pic8/18P-11w	freshwater	North America	USA: Picnic Pond, Minnesota
3	AY195972	AS-29	freshwater	North America	USA: North Dakota, Arrowwood Lake, Arrowwood National Wildlife Refuge
4a	MT423987	SAG 17.98	freshwater	Europe	Germany: Göttingen, basin in warm greenhouse in Botanical Garden
4b	LC472538	NIES-4292	freshwater	Asia	Japan: Lake Kasumigaura, Ibaraki
4b	AB488587	NIES-2342	freshwater	Asia	Japan: Lake Otadomarinuma, Hokkaido
5	MT423986	SAG 251-2	freshwater	Europe	Switzerland: Schiern/Bern, static water tank
5	AY195970	AS 5-1	freshwater	North America	USA: North Dakota, Arrowwood Lake, Arrowwood National Wildlife Refuge

Haplotype	V9	strain/clone/OTU	habitat	region	origin
1a	FN298929	SAG 211-40c	symbiont	North America	USA: Massachusetts, Manumet Beach, endosymbiont from Spongilla lacustris
1a	MT423984	SAG 211-40b	symbiont	North America	USA: Massachusetts, Manumet Beach, endosymbiont from Ephydatia fluviatilis
1a	MT423985	SAG 251-1	freshwater	North America	Canada: Quebec, Lac Grandpré
1a	FN298930	UTEX 838	symbiont	North America	USA, endosymbiont from Ephydatia fluviatilis
1a	MK021422	OTU1033	marine	Europe	Netherlands: Texel, intertidal mudflat sediment
1a	*MK988745	OTU_2476	freshwater	Europe	* see Metz et al. (2019)
1a	GU067789	ESS220206.010	freshwater	Europe	Luxembourg: Lake Esch sur Sure
1a	FJ153668	GoC3_B04	marine	Europe	anoxic Gotland Deep (Baltic Sea) water sample
1a	FJ153652	GoC2_D01	marine	Europe	anoxic Gotland Deep (Baltic Sea) water sample
1a	AY197623	MDL1/12-8	freshwater	North America	USA: North Dakota, Mud Lake, Arrowwood National Wildlife Refuge
1a	AB109544	OL2-1	symbiont	Asia	USA: Florida, Orlando, ditch, endosymbiont of Paramecium bursaria
1a	AY220082	MDL5-9	freshwater	North America	USA: North Dakota, Mud Lake, Arrowwood National Wildlife Refuge
1a	AY195975	Itas 9/21 14-5w	freshwater	North America	USA: Minnesota, Lake Itasca
1a	AB080305	SAG 14.87	freshwater	Europe	United Kingdom: River Crouch
1a	KX139550	isolate HB-2	symbiont	Asia	China: Hubei, Lake Ziyang, endosymbiont of Spongilla sp.
1a	KX139549	isolate HB-1	symbiont	Asia	China: Hubei, Lake Ziyang, endosymbiont of Spongilla sp.
1a	KX139548	isolate TB-2	symbiont	Asia	China: Tibet, Nagtsang Tso, endosymbiont of Spongilla sp.
1a	KX139547	isolate TB-1	symbiont	Asia	China: Tibet, Nagtsang Tso, endosymbiont of Spongilla sp.
1b	AH012393	3D4	freshwater	Europe	Switzerland: Lake Alpnach
1b	AH012392	1A1	freshwater	Europe	Switzerland: Lake Hagel
1b	AF357149	4A3	freshwater	Europe	Switzerland: Lake Alpnach
1c	FJ153657	GoC2_E07	marine	Europe	anoxic Gotland Deep (Baltic Sea) water sample
1d	AB488584	NIES-2335	freshwater	Asia	Japan: Lake Kuttara, Hokkaido
2	AY197629	Pic8/18P-11w	freshwater	North America	USA: Picnic Pond, Minnesota
3	AY195972	AS-29	freshwater	North America	USA: North Dakota, Arrowwood Lake, Arrowwood National Wildlife Refuge
4	MT423987	SAG 17.98	freshwater	Europe	Germany: Göttingen, basin in warm greenhouse in Botanical Garden
4	*MK988949	OTU_17944	freshwater	South America	* see Metz et al. (2019)
4	LC472538	NIES-4292	freshwater	Asia	Japan: Lake Kasumigaura, Ibaraki
5	MT423986	SAG 251-2	freshwater	Europe	Switzerland: Schiern/Bern, static water tank

Haplotype	V9	strain/clone/OTU	habitat	region	origin
5	AY195970	AS 5-1	freshwater	North America	USA: North Dakota, Arrowwood Lake, Arrowwood National Wildlife Refuge
5	MK021894	OTU_0766	marine	Europe	Netherlands: Texel, mud sediment
5	*MK988718	OTU_104555	*	*	* see Metz et al. (2019)
5	*MK988834	OTU_105582	*	*	* see Metz et al. (2019)
5	*MK988899	OTU_106600	*	*	* see Metz et al. (2019)
5	*MK988900	OTU_106601	*	*	* see Metz et al. (2019)
5	*MK988901	OTU_106602	*	*	* see Metz et al. (2019)
5	*MK988902	OTU_106603	*	*	* see Metz et al. (2019)
5	*MK988903	OTU_106604	*	*	* see Metz et al. (2019)
5	*MK988904	OTU_106605	*	*	* see Metz et al. (2019)
5	*MK988906	OTU_106607	*	*	* see Metz et al. (2019)
5	*MK988907	OTU_106608	*	*	* see Metz et al. (2019)
5	*MK988937	OTU_115044	*	*	* see Metz et al. (2019)
5	*MK989026	OTU_117783	*	*	* see Metz et al. (2019)
5	*MK989027	OTU_117786	*	*	* see Metz et al. (2019)
5	*MK989028	OTU_117787	*	*	* see Metz et al. (2019)
5	*MK989029	OTU_117788	*	*	* see Metz et al. (2019)
5	*MK989089	OTU_132700	*	*	* see Metz et al. (2019)
5	*MK989149	OTU_133090	*	*	* see Metz et al. (2019)
5	*MK989150	OTU_133091	*	*	* see Metz et al. (2019)
5	*MK989151	OTU_133092	*	*	* see Metz et al. (2019)
5	*MK989153	OTU_133103	*	*	* see Metz et al. (2019)
5	*MK989202	OTU_152939	*	*	* see Metz et al. (2019)
5	*MK989251	OTU_153545	*	*	* see Metz et al. (2019)
5	*MK989252	OTU_153546	*	*	* see Metz et al. (2019)
5	*MK989253	OTU_153547	*	*	* see Metz et al. (2019)
5	*MK989254	OTU_153548	*	*	* see Metz et al. (2019)
5	*MK989255	OTU_153551	*	*	* see Metz et al. (2019)
5	*MK989257	OTU_153589	*	*	* see Metz et al. (2019)

Haplotype	V9	strain/clone/OTU	habitat	region	origin
5	***MK989276**	OTU_158735	*	*	* see Metz et al. (2019)
5	***MK989278**	OTU_158958	*	*	* see Metz et al. (2019)
5	***MK988736**	OTU_16121	*	*	* see Metz et al. (2019)
5	***MK988737**	OTU_16283	*	*	* see Metz et al. (2019)
5	***MK988738**	OTU_16284	*	*	* see Metz et al. (2019)
5	***MK988742**	OTU_16445	*	*	* see Metz et al. (2019)
5	***MK988945**	OTU_17330	*	*	* see Metz et al. (2019)
5	***MK988948**	OTU_17533	*	*	* see Metz et al. (2019)
5	***MK989286**	OTU_176522	*	*	* see Metz et al. (2019)
5	***MK989321**	OTU_178099	*	*	* see Metz et al. (2019)
5	***MK988950**	OTU_17945	*	*	* see Metz et al. (2019)
5	***MK988954**	OTU_18353	*	*	* see Metz et al. (2019)
5	***MK988955**	OTU_18354	*	*	* see Metz et al. (2019)
5	***MK989381**	OTU_184771	*	*	* see Metz et al. (2019)
5	***MK989382**	OTU_184775	*	*	* see Metz et al. (2019)
5	***MK989383**	OTU_184779	*	*	* see Metz et al. (2019)
5	***MK989384**	OTU_184781	*	*	* see Metz et al. (2019)
5	***MK989054**	OTU_19295	*	*	* see Metz et al. (2019)
5	***MK989061**	OTU_19789	*	*	* see Metz et al. (2019)
5	***MK989064**	OTU_20025	*	*	* see Metz et al. (2019)
5	***MK989065**	OTU_20026	*	*	* see Metz et al. (2019)
5	***MK989068**	OTU_20271	*	*	* see Metz et al. (2019)
5	***MK989078**	OTU_21084	*	*	* see Metz et al. (2019)
5	***MK989173**	OTU_21983	*	*	* see Metz et al. (2019)
5	***MK989176**	OTU_22933	*	*	* see Metz et al. (2019)
5	***MK989185**	OTU_24011	*	*	* see Metz et al. (2019)
5	***MK989187**	OTU_24434	*	*	* see Metz et al. (2019)
5	***MK989287**	OTU_25270	*	*	* see Metz et al. (2019)
5	***MK989288**	OTU_25271	*	*	* see Metz et al. (2019)

Haplotype	V9	strain/clone/OTU	habitat	region	origin
**5**	***MK989289**	OTU_25274	*	*	* see Metz et al. (2019)
**5**	***MK989294**	OTU_26652	*	*	* see Metz et al. (2019)
**5**	***MK989296**	OTU_26750	*	*	* see Metz et al. (2019)
**5**	***MK989301**	OTU_27634	*	*	* see Metz et al. (2019)
**5**	***MK989303**	OTU_28003	*	*	* see Metz et al. (2019)
**5**	***MK989305**	OTU_28077	*	*	* see Metz et al. (2019)
**5**	***MK989306**	OTU_28692	*	*	* see Metz et al. (2019)
**5**	***MK989307**	OTU_28695	*	*	* see Metz et al. (2019)
**5**	***MK989429**	OTU_29939	*	*	* see Metz et al. (2019)
**5**	***MK989432**	OTU_30533	*	*	* see Metz et al. (2019)
**5**	***MK989433**	OTU_30535	*	*	* see Metz et al. (2019)
**5**	***MK989434**	OTU_31180	*	*	* see Metz et al. (2019)
**5**	***MK989435**	OTU_31182	*	*	* see Metz et al. (2019)
**5**	***MK989442**	OTU_32545	*	*	* see Metz et al. (2019)
**5**	***MK989444**	OTU_33336	*	*	* see Metz et al. (2019)
**5**	***MK989448**	OTU_34174	*	*	* see Metz et al. (2019)
**5**	***MK989449**	OTU_34870	*	*	* see Metz et al. (2019)
**5**	***MK989453**	OTU_35850	*	*	* see Metz et al. (2019)
**5**	***MK989454**	OTU_35921	*	*	* see Metz et al. (2019)
**5**	***MK988695**	OTU_89777	*	*	* see Metz et al. (2019)
**5**	***MK988696**	OTU_89778	*	*	* see Metz et al. (2019)
**5**	***MK988697**	OTU_89779	*	*	* see Metz et al. (2019)
**5**	***MK988698**	OTU_89780	*	*	* see Metz et al. (2019)
**5**	***MK988701**	OTU_89783	*	*	* see Metz et al. (2019)
**5**	***MK988702**	OTU_89784	*	*	* see Metz et al. (2019)
**5**	***MK988749**	OTU_95557	*	*	* see Metz et al. (2019)
**5**	***MK988813**	OTU_97095	*	*	* see Metz et al. (2019)
**5**	***MK988814**	OTU_97097	*	*	* see Metz et al. (2019)
**5**	***MK988828**	OTU_99060	*	*	* see Metz et al. (2019)

Haplotype	ITS2	strain/clone/OTU	habitat	region	origin
**1a**	**FN298929**	SAG 211-40c	symbiont	North America	USA: Massachusetts, Manumet Beach, endosymbiont from Spongilla lacustris
**1a**	**KX139558**	isolate HB_4	symbiont	Asia	China: Hubei, Lake Ziyang, endosymbiont of Spongilla sp.
**1a**	**KX139557**	isolate HB_3	symbiont	Asia	China: Hubei, Lake Ziyang, endosymbiont of Spongilla sp.
**1a**	**KX139556**	isolate HB_2	symbiont	Asia	China: Hubei, Lake Ziyang, endosymbiont of Spongilla sp.
**1a**	**KX139555**	isolate HB_1	symbiont	Asia	China: Hubei, Lake Ziyang, endosymbiont of Spongilla sp.
**1a**	**KX139554**	isolate TB_4	symbiont	Asia	China: Tibet, Nagtsang Tso, endosymbiont of Spongilla sp.
**1a**	**KX139553**	isolate TB_3	symbiont	Asia	China: Tibet, Nagtsang Tso, endosymbiont of Spongilla sp.
**1a**	**KX139552**	isolate TB_2	symbiont	Asia	China: Tibet, Nagtsang Tso, endosymbiont of Spongilla sp.
**1a**	**KX139551**	isolate TB_1	symbiont	Asia	China: Tibet, Nagtsang Tso, endosymbiont of Spongilla sp.
**1a**	**MT423984**	SAG 211-40b	symbiont	North America	USA: Massachusetts, Manumet Beach, endosymbiont from Ephydatia fluviatilis
**1a**	**FN298930**	UTEX 838	symbiont	North America	USA, endosymbiont from Ephydatia fluviatilis
**1b**	**MT423985**	SAG 251-1	freshwater	North America	Canada: Quebec, Lac Grandpré
**1b**	**KY932451**	OTU55	others	unknown	human
**1b**	**KY932180**	OTU74	others	unknown	human
**1b**	**KY932073**	OTU147	others	unknown	human
**1b**	**KY934998**	OTU514	others	unknown	human
**1c**	**KP212163**	F106U1B1	freshwater	Europe	Ukraine: Lviv, Jaworiw, Dobrostany
**1c**	**KP212162**	F107U1A2	freshwater	Europe	Ukraine: Lviv, Jaworiw, Dobrostany
**1c**	**KP212161**	F107U1B1	freshwater	Europe	Ukraine: Lviv, Jaworiw, Dobrostany
**4a**	**MT423987**	SAG 17.98	freshwater	Europe	Germany: Göttingen, basin in warm greenhouse in Botanical Garden
**4b**	**MH364432**	OTU_314	freshwater	Asia	China: seagrass rhizosphere
**5**	**MT423986**	SAG 251-2	freshwater	Europe	Switzerland: Schiern/Bern, static water tank
